# Decoding Evolution of Rubioideae: Plastomes Reveal Sweet Secrets of Codon Usage, Diagnostides, and Superbarcoding

**DOI:** 10.3390/genes15050562

**Published:** 2024-04-27

**Authors:** Kamil Ciborowski, Monika Szczecińska, Mateusz Maździarz, Jakub Sawicki, Łukasz Paukszto

**Affiliations:** Department of Botany and Evolutionary Ecology, University of Warmia and Mazury in Olsztyn, Plac Łódzki 1, 10-727 Olsztyn, Poland; kamil.ciborowski.1@student.uwm.edu.pl (K.C.); monika.szczecinska@uwm.edu.pl (M.S.); mateusz.mazdziarz@uwm.edu.pl (M.M.); lukasz.paukszto@uwm.edu.pl (Ł.P.)

**Keywords:** diagnostides, chloroplast, plastome, *Galium*, Rubiaceae, superbarcoding

## Abstract

*Galium* genus belongs to the Rubiaceae family, which consists of approximately 14,000 species. In comparison to its well-known relatives, the plastomes of the *Galium* genus have not been explored so far. The plastomes of this genus have a typical, quadripartite structure, but differ in gene content, since the *infA* gene is missing in *Galium palustre* and *Galium trfidum*. An evaluation of the effectiveness of using entire chloroplast genome sequences as superbarcodes for accurate plant species identification revealed the high potential of this method for molecular delimitation within the genus and tribe. The *trnE*-UUC—*psbD* region showed the biggest number of diagnostides (diagnostic nucleotides) which might be new potential barcodes, not only in *Galium*, but also in other closely related genera. Relative synonymous codon usage (RSCU) appeared to be connected with the phylogeny of the Rubiaceae family, showing that during evolution, plants started preferring specific codons over others.

## 1. Introduction

The Rubiaceae family is found on almost all continents. It consists of about 611 genera that include around 14,000 species [1,2]. These species include all forms of vascular plants: trees and shrubs such as *Cinchona officinalis* or *Coffea arabica*, herbaceous perennials (e.g., *Galium verum*) that dominate other forms, and annual herbs (e.g., *Galium aparine*). The great diversity of this very interesting family has made it a target for research in many fields, such as biochemistry, medicine, ecology, and phylogenetics. This has led to numerous studies conducted by taxonomists all over the world. For example, a very interesting feature is the difference in the number of P-leaves in leaf whorls between Rubiaceae genera [3]. Because of these differences, *Limnosipanea ternifolia* was thought to be a separate species from *Limnosipanea sprucea* because of the smaller number of leaves in a whorl [4]. Nowadays, *L. ternifolia* is considered the same species as *L. sprucea*. This situation is evidence that morphological classification is sometimes insufficient. The alternative way to explore taxonomic and phylogenetic relationships among Rubiaceae is to study bioactive compounds. Plants included in this family are rich in secondary metabolites (e.g., *C. arabica* produces caffeine) that can be used in chemotaxonomic research. Biochemical compounds, distributed between different taxonomic groups, have shown a correlation that supports the evolutionary theory that Rubioideae is the oldest subfamily, followed by Ixoroideae and then Cinchonoideae [5]. Another method used in taxonomic studies is molecular characteristics, interspecific and intraspecific differences, distributed across the plastome. The use of single-nucleotide polymorphisms (SNPs), indels (insertions/deletions), rearrangements, and translocations increases the possibility of carrying out studies on phylogenetics, barcode analysis, species recognition, population genetics, or endangered species conservation [6]. Nowadays, many molecular studies employ chloroplast DNA (cpDNA), including work on the family Rubiaceae, especially the subfamily Ixoroideae, to which the coffee tree belongs. For example, plastome structural variations (PSVs) appeared to be very abundant among the Coffeae alliance tribes [7]. To sum up, it is crucial for phylogenetic scientists to explore the genetic structures of plants.

The Rubioideae subfamily is the richest group of species, with around 8000 species, followed by Ixoroideae with around 4000 species and Cinchonoideae with 1700 species. However, studies on phylogenetics based on complete chloroplast genomes for this subfamily are still rare [6]. The Rubieae tribe, which includes around 1000 species (most genera) from the subfamily Rubioideae, is distributed worldwide and is common in various habitats, ranging from cold temperate regions to tropical forests [8]. Early molecular analyses focused on only one or few regions in the plastid genome [9]. These studies have shown a problematic characteristic of the Rubioideae subfamily, in that it contains many non-monophyletic relationships. For example, *Galium* species create a common clade with *Asperula* species, although they are morphologically different.

Advances in DNA sequencing technology have provided scientists with a high-efficiency and cost-effective method of obtaining complete chloroplast genome sequences, which are typically inherited uniparentally, lack recombination, and are compact in size. Plastomes, in contrast to plant mitogenomes, have a stable structure, at least at the family level, with the rare presence of heteroplasmy and horizontally transferred regions [10,11].

The use of complete plastome sequences can significantly improve the resolution at lower taxonomic levels in plant phylogeny, phylogeography, and population genetics [12]. The application of whole-chloroplast genome sequences as superbarcodes for plant species identification has emerged as a powerful tool in the field of plant taxonomy and biodiversity studies. It can be used to differentiate species or varieties and to identify admixtures as well as field contaminants [13,14]. However, besides crops and other industrially important species of Rubiaceae, some genera have poorly developed genetic resources.

These advances in technology are significant for many plant groups, such as the tribe Rubieae, in fields such as ecology and plant conservation. *G. trifidum* is an endangered species in Poland [15]. While *G. palustre* is a very common species for wetlands in Central and East Europe, it can be easily mistaken for *G. trifidum* because these species are very similar. Actually, the recognition of these species is only possible when flowers are developed. Superbarcodes might be very helpful for resolving these kinds of problems because new technology allows for rapid and efficient sequencing, which may aid scientists in species recognition.

Therefore, during our research, chloroplast genomes of *Galium* species, which have never been published before, were sequenced and assembled. This is the first work which embraces most complete chloroplast sequences of *Galium* species, including two isolates of *G. trifidum*, two isolates of *G. palustre*, one of *Galium odoratum*, and one of *G. verum*. We aimed at the characterisation of plastomes of Rubioideae and the identification of the most efficient loci in the plastomes for resolving phylogenetic relationships at lower taxonomic levels within this clade, and tested for an association between relative synonymous codon usage (RSCU) and the evolution of Rubiaceae. Newly assembled sequences and data available online aided in the comparison of plastomes across the whole Rubieae tribe, allowed for the identification of specific genomic regions as the main sources of diagnostic nucleotides, and indicated a connection between RSCU and the phylogeny of the Rubiaceae family.

## 2. Materials and Methods

### 2.1. DNA Extraction

Total genomic DNA was extracted from leaf tissue added to silica gel using the DNeasy^®^ Plant Mini Kit (Qiagen, Hilden, Germany). Stems were ground with silica beads using a MiniBead-Beater tissue disruptor for 50 s, and they were subsequently processed using the manufacturer’s protocol. DNA quantity was estimated with a Qubit fluorometer system (Invitrogen, Carlsbad, NM, USA) using a Quant-IT ds-DNA BR Assay Kit (Invitrogen). For the library construction of *G. trifidum*, previously extracted DNA was used [16].

### 2.2. Library Preparation and Assembly

The genomic library was constructed with a TruSeq Nano DNA kit (Illumina, San Diego, CA, USA) and was sequenced using HiSeqX (Illumina) to generate 150 bp paired-end reads at Macrogen Inc. (Seoul, Republic of Korea) with a 350 bp insert size between paired ends. The raw reads were evaluated by a Trimmomatic 0.39 tool [17] in the quality check process with following parameters: PHRED score > 20, a 150 bp trimmed sequence length, number of Ns < 1%. The filtered reads were transferred to the de novo plastome assembly of two *G. trifidum* isolates (ID1 and ID2) and two *G. palustre* isolates (ID1 and ID2). The assembly was performed by NOVOPlasty 4.3 software [18] with default settings and *rbc*L gene as the seed. The NOVOPlasty software enables the de novo assembly of short circular genomes. Meanwhile, raw reads of *G. odoratum* and *G. verum* were downloaded from the Sequence Read Archive (SRA) and also assembled with NOVOPlasty (Table S1). In the next steps, the assembly of all plastomes was verified manually in Geneious Prime 2023.2.1 (Biomatters, Auckland, New Zealand), which allowed us to conduct a comprehensive sequence analysis (visualisation, alignment, annotation editing, and mapping). The circular sequence of each genome was remapped by the Geneious Prime 2023.2.1 mapping algorithm with custom parameters (minimum overlap = 80 bp and minimum overlap identity = 96%). After full plastome completion, the sequences were annotated according to *G. aparine* (NC_036969.1; chloroplast NCBI record) with the support of the Transfer Annotation option in the Geneious Prime 2023.2.1 software. 

### 2.3. Chloroplast Genome Visualisation, Phylogenetic Analysis, Nucleotide Diversity, and Species Delimitation

The chloroplast genome of the selected representative of the *Galium* genus (NC_082337) was visualised in the organellar genome map drawer—the OGDRAW 1.3.1 web tool [19]. The three representants of the *Galium* (NC_082337), *Rubia* (NC_047470), and *Leptodermis* (NC_049160) genera were compared to obtain details about the boundaries, length, and structure of large single-copy regions (LSCs), small single-copy regions (SSCs), and inverted repeat (IR) regions using Irplus 1.0 [20]. The reason why only single representatives were used in Irplot was the high similarity of the LSC, SSC, and Irs structures within each genus.

The phylogenetic analysis was carried out on 44 chloroplast genomes from Rubiaceae tribes and *Exacum aphine* belonging to the Gentianaceae family (Table S2). The phylogenetic tree was calculated using the Maximum Likelihood (ML) method with the TVM+F+I+G4 model chosen according to the Bayesian Information Criterion (BIC). The whole process was conducted using the Phylogenetic and Molecular Evolution (PhaME) pipeline [21]. The MAFFT (File S1) alignment used for the PhaME pipeline was created in Geneious Prime 2023.2.1 with the following parameters: automatic algorithm, scoring matrix: 200 PAM/k = 2, gap open penalty: 1.53, offset value: 0.123 [22]. The final view of the phylogenetic tree was created with the ggtree 3.10.1 [23] and ggplot2 3.5.1 [24] R environment packages [25]. Using these packages, RSCU (relative synonymous codon usage) was added on the right side of the phylogenetic tree. The aim of this procedure was to visualise changes in RSCU values for GCC, GCT, TTC, GGT, GGG, and TAG codons between the analysed species. Statistical differences between the tribes Rubieae, Paederieae, and Morindeae for codons were calculated with Kruskal–Wallis test and confirmed with a post hoc Dunn test (Table S3). A divergence time tree was created in Mega11 11.0.13 software [26]. The phylogenetic tree generated during this research and 10 calibration constraints were used to calculate the time tree with the RelTime method [27,28]. Calibration constraints were taken from the published article [29]. The visualisation was carried out in the ape 5.7-1 and strap 1.6-0 R packages [30,31].

For diversity insight within the Rubieae tribe, two parameters were estimated—diagnostic nucleotides and nucleotide diversity (π). The investigation was performed based on MAFFT alignments generated in Geneious Prime 2023.2.1 software. The spider 1.5.0 R package was applied to calculate diagnostides between *Galium*, *Rubia*, and *Leptodermis* species [32]. Next, nucleotide diversity was estimated to describe less or more divergent regions within the plastome genome using the PopGenome 2.7.7 R package [33]. Both estimators (diagnostic nucleotide and π) were calculated in a 500 bp frameshift window. The computations of both divergence parameters were focused on the LSC (long single-copy), SSC (short single-copy), and only one of the IR (inverted repeat) regions. Additionally, the SNP (single-nucleotide polymorphism) and indel (insertion/deletion) variants were identified based on the *Galium* genus alignment using the Variant Calling subprogram in Geneious Prime 2023.2.1 software (with cut-off, *p*-value < $10\times 10^{-7}$). Finally, all divergence variables were visualised in a Circos plot [34].

### 2.4. Relative Synonymous Codon Usage

The *seqinr* 4.2 R package was used to calculate the RSCU, which is considered to be the ratio of the observed codon frequency to the expected frequency that would be observed if all synonymous codons for a given amino acid were used with equal frequency [35]. To determine the differences in codon usage between genera, the RSCU values were calculated separately for each genus and presented together using the ggplot2 3.5.1 package in the form of a bar plot. The ComplexHeatmap 2.18.0 library was used to plot the heatmap from the RSCU values for each codon in all species used in the investigation [36].

## 3. Results

### 3.1. Characteristics of Galium Chloroplast Genomes

**Figure 1:**
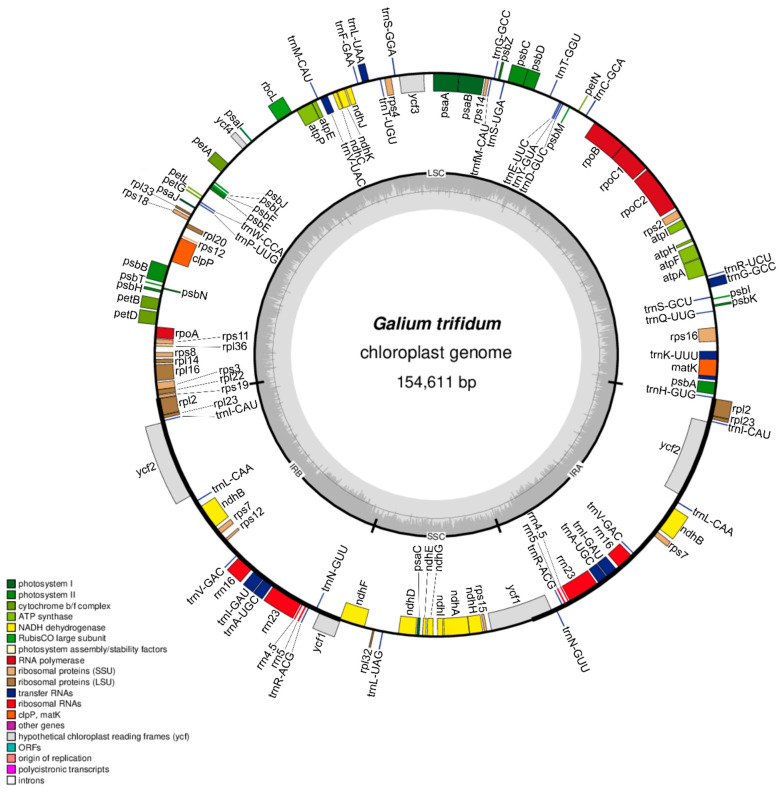
Map of *G. trifidum* 1 genome. The legend indicates functions and names of genes visible on inner and outer sides of the circle. The smaller circle shows the quadripartite structure of the chloroplast genome with the GC content presented as the bar plot. Introns are not pointed out.

The chloroplast genome of *G. trifidum* 1 (Figure 1, Tables S1 and S2) is 154,611 bp long and contains four regions typical of most vascular plants [37]. The large single-copy (LSC) region is 84,976 bp long, the small single-copy (SSC) region is 17,127 bp long, and inverted repeat regions (IRA and IRB) are 26,254 bp long (Table S3). The LSC contains 81 genes, of which 59 genes are protein-coding and 22 are tRNA-coding. The SSC region includes 12 genes—11 protein and 1 tRNA coding. Inverted repeat region A has five protein-coding genes, seven tRNA-coding genes, and four rRNA genes. In the case of inverted repeat region B, there is the same number of rRNA and tRNA genes as in IRA. However, IRB harbours eight protein-coding genes. It is interesting to note that both the IRB and IRA regions share two copies of the *ycf1* gene with the SSC region, and the shorter one is a pseudogene (Figure S1). The *ndhF* gene marks the boundary between IRB and SSC, while the *rps19* gene marks the boundary between IRB and LSC. The assembled plastomes of *G. trifidum* 2, *G. palustre* 1, and *G. palustre* 2 have an identical structure and the same number of genes as the described genome. *G. verum* and *G. odoratum* have one additional protein-coding gene (*infA*). All newly assembled sequences have a GC content of around 37% (*G. trifidum* and *G. palustre*: 37%, *G. verum*: 37.2%, *G. odoratum*: 37.3%). Generally, the chloroplast genome contains 81 protein-coding genes, 30 transfer RNA genes, and 4 rRNA genes, while 18 genes are duplicated in the IR regions.

**Figure 2:**
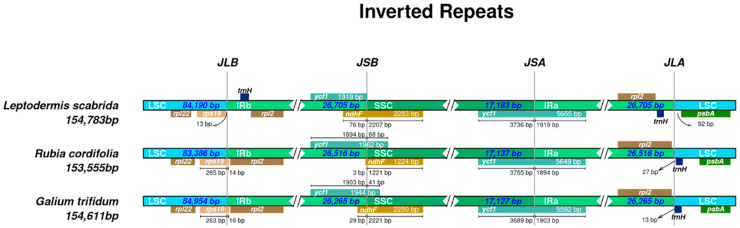
Irplot presenting junction sites of three different species. JLB, JSB, JSA, and JLA are abbreviations for the following boundaries: junction between LSC and IRB, SSC and IRB, SSC and IRA, and LSC and IRA, respectively. Each species is a representative of a different genus.

Visualising the boundaries of chloroplast genome junction sites can reveal general differences between species or genera (Figure 2). In the case of *Leptodermis scabrida*, junction site B (between the LSC and IRB regions) is located 13 bp from the *rps19* gene located in LSC, while in the remaining sequences, the boundary is located within the *rps19* gene. Another distinction is observed between the IRB and SSC regions in *L. scabrida* where the boundary is located within the *ndhF* gene (76 bp belongs to the IRB region and 2207 bp to the SSC). In *G. trifidum*, JSB (the junction site between IRB and SSC) is located 29 bp from the beginning of the *ndhF* gene, while in *Rubia cordifolia*, it is only 3 bp from the start. In the case of *L. scabrida*, JSB is located outside of the *ycf1* gene. It is worth noting that the *trnH* gene in *L. scabrida* is located in the IR regions. The *trnH* gene of the remaining species is located in the LSC region, 27 bp from the JLA (junction site between LSC and IRA regions) in the case of *R. cordifolia*, and 13 bp in the case of *G. trifidum*.

### 3.2. Intraspecific Variation of Plastid Genomes of Galium, Leptodermis, and Rubia

**Figure 3:**
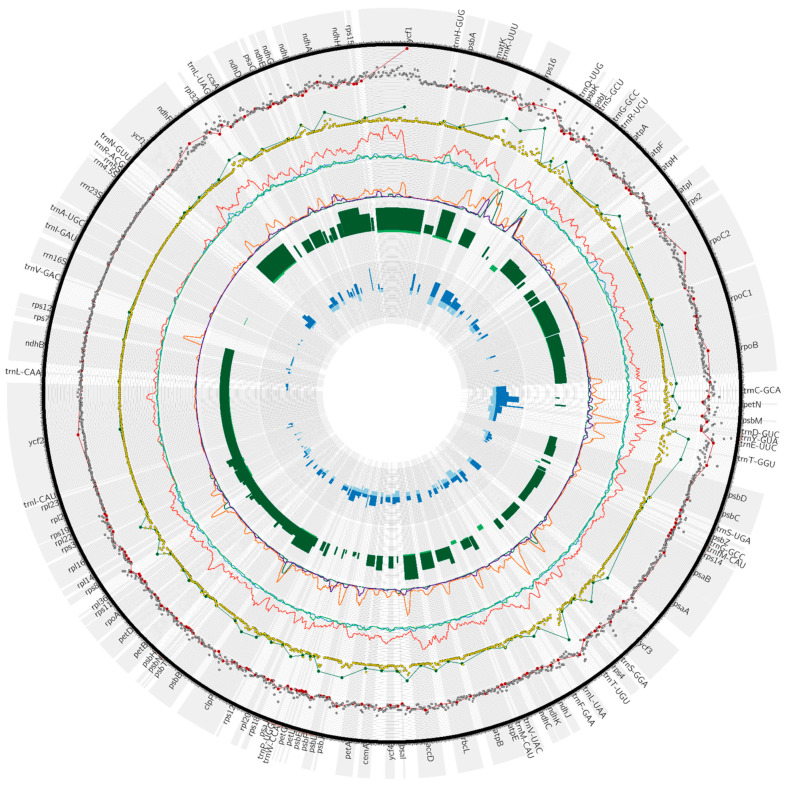
Comparisons of the collection of data of *Galium* species plastomes for three different genera. In the first plot (the outermost layer beneath gene names) are presented SNPs for eight *Galium* species (grey scatter plot—number of SNPs per 100 bp, red—number of SNPs for genic and intergenic regions). The second layer presents indels that are labelled as yellow dots—number of indels per 100 bp—and green dots—number of indels in genic and intergenic regions. The next two layers show nucleotide diversity (*Galium*—red, *Leptodermis*—blue, *Rubia*—light green) and the number of diagnostides (*Galium*—orange, *Leptodermis*—purple, *Rubia*—dark green), respectively. The first histogram contains information about the percentage of SNPs (dark green) and indels (light green) in coding regions. The second histogram shows the percentage of SNPs (dark blue) and indels (light blue) in noncoding regions.

The biggest aggregations of SNPs can be found in two regions: one between *rps16* and *trnQ*-UUG (192) and the other within *ycf1* (426), situated in the SSC region (Figure 3, Tables S4 and S5). In the case of indels, these type of changes are mostly concentrated in the vicinity of noncoding regions: *trnK*-UUU—*rps16* (61), *rps16*—*trnQ*-UUG (81), *trnE*-UUC—*trnT*-GGU (67), and *trnT*-UGU—*trnL*-UAA (76) (Tables S6 and S7). Upon examining the nucleotide diversity (π diversity) plots, it is clear that *Galium* exhibits significantly higher values than the other two genera. The highest values are located in *ycf1* in SSC (0.0052), while the second highest value is created by two peaks that are very close to each other between *trnD*-GUC and *trnE*-UUC (0.0048) (Table S8). In the case of *Rubia*, the most characteristic peak is located at the beginning of the sequence, in the *psbA* gene (0.004). *Leptodermis* species have significantly lower values in comparison to *Galium* and *Rubia*, with the highest point in *rps2* (0.0016). The percentage values of SNPs and indels between *Galium* species look as follows: the *matK* gene has approximately 7.3% of SNPs which is the highest value among all coding regions (Table S9). The intron of *trnG*-GCC has the highest percentage of indels, at approximately 1.3% (Table S10). The noncoding region with the highest percentage of SNPs is the spacer *trnD*-GUC—*trnY*-GUA (almost 9.5%), and it is worth noting that this region is very short (Table S11). The *trnE*-UUC—*trnT*-GGU region includes approximately 6.6% of indels, which is the highest value in noncoding regions (Table S12). The plots show little differentiation in the IR region (from *rps19* to *ndhF*), although there is one orange peak located in the *trnI*-GAU gene.

### 3.3. Molecular Delimitation of Galium, Leptodermis, and Rubia

One method used in molecular taxonomy involves defining diagnostic characteristics in specific regions between species. To retrieve the necessary data, diagnostides (a new name for diagnostic nucleotides) should be identified across the studied plastomes. We calculated diagnostides for each genus to find regions with high potential for phylogenetic analyses of Rubioideae at shallow evolutionary scales (Figure 3, Table S13). *Galium* species showed the highest peak in the *trnE*-UUC—*psbD* region (52), followed by a peak in the *rps16* (51) and *rps16*—*trnQ*-UUG noncoding region (50). The *Rubia* species has surprisingly high values in the leading region, *rps16*—*trnQ*-UUG (139). In *Leptodermis*, the location with the highest abundance of diagnostides is between *trnS*-GCU and *trnG*-GCC (37), while in *Rubia*, it is the second highest peak (129). Moreover, the *trnS*-GCU—*trnG*-GCC region is the only location where *Galium*, *Rubia*, and *Leptodermis* possess relatively high value, which means that the intergenic region can delimitate the intrageneric relations of these three members. Other interesting regions include *rps4*, *psbB*—*psbT*, and *rps3*—*rpl22*, where *Galium* and *Rubia* generated similar peaks.

### 3.4. Codon Usage

**Figure 4:**
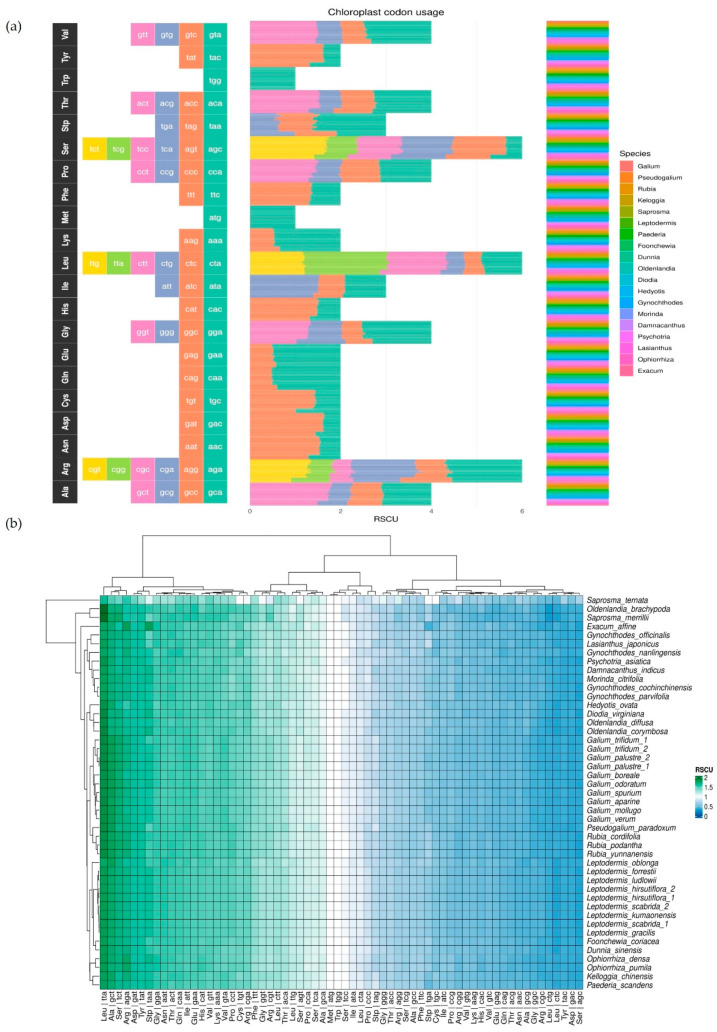
(**a**) The Relative synonymous codon usage (RSCU) plot. For every amino acid, a nucleotide triplet found in our analysis is implemented. In the middle is presented a codon usage (each codon has a different colour) summed for each genus used in this research with genera listed alongside. (**b**) The RSCU heatmap shows species preference of codon usage: dark blue and light blue colours represent less preferred codons, white colour represents codons that are neither less preferred nor more preferred, and light green and dark green colours represent more preferred codons. The left side of the figure (*y* axis) shows phylogenetic relationships, and the upper side of the figure (*x* axis) shows RSCU relationships.

The relative synonymous codon usage (RSCU) plot (Figure 4a, Table S14) shows preferences of codon usage in different genera. Clear changes are observed, e.g., in valine (Val), where the GTA codon is more preferred than the GTC codon in *Galium* and *Pseudogalium* species. This trend is slightly different in other genera, especially *Exacum*, *Ophiorrhiza*, *Lasianthus*, and *Psychotria*, where they clearly use more GTC codons than *Galium* and *Pseudogalium*, simultaneously decreasing the number of GTA codons in exons. On the other hand, the RSCU heatmap (Figure 4b) shows that valine has similar GTA usage across all species included in the analysis, which may indicate that this difference is not significant. The heatmap is useful for presenting the preference of some triplet usages that might be overlooked in Figure 4a. For example, most species prefer the TTA codon for leucine (Leu) over other codons, as shown in the heatmap. *Oldenladnia brachypoda* and *Saprosma merrilli* are particularly prominent in their usage of TTA. Additionally, plants belonging to the Rubieae tribe exhibit a greater preference for TTA than those in the Paederieae tribe.

### 3.5. Phylogenetic Analysis

**Figure 5:**
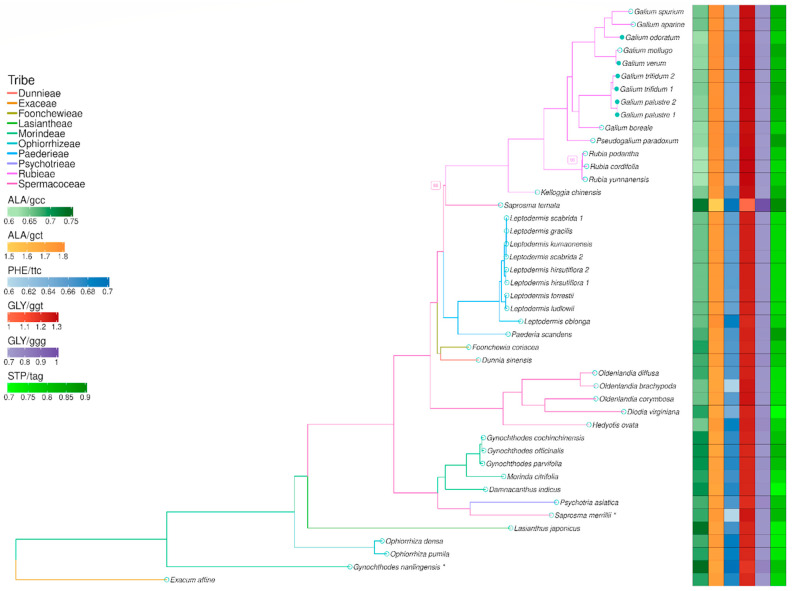
Maximum Likelihood (ML) phylogenetic tree based on analysed chloroplast genomes. Bootstrap values lower than 100 are present beside nodes. Branch colours indicate a tribe (legend placed in the left upper corner of the figure). Blue filled dots mean that plastomes of these species were assembled in this research. “*” means that these species might be non-monophyletic. Heatmap on the right shows the RSCU, and all codons with scales taken into account are listed on the left side of the figure.

Our phylogenetic tree is divided into 10 tribes (Figure 5). *Exacum affine*, belonging to the Exaceae tribe, was chosen as an outgroup. This tribe belongs to the Gentianaceae family, and the rest of the species belong to the Rubiaceae family. Only two nodes have bootstrap values below 100: the node connecting *Saprosma ternata* and the Rubieae tribe (88) and the node connecting *Rubia podantha* and *R. cordifolia* (96). Furthermore, *S. merrillii* is not grouped with *Saprosma ternata*, while *Gynochthodes nanlingensis* groups far away from other *Gynochthodes* species. An additional analysis was performed by mapping sequences of genes available in the National Center of Bioinformatics (NCBI) database to both *S. merrillii* and *S. ternata* (*trnH*—*psbA*, *matK*, *rps16*, *trnS*—*trnG*, *rpoB*—*trnC*, *trnL*, *rbcL*, *ndhF*). The comparison revealed moderate differences between *S. merrillii* and the mapped regions, whereas in *S. ternata*, only few substitutions were observed. This observation may explain the unexpected localisation of *S. merrillii* taxa on the phylogenetic tree. To validate *G. nanlingensis*, sequences of the marker genes *matK*, *rbcL*, and *ndhF* were extracted and mapped to the reference genome of *Gynochthodes parviflora* (NC_054151), along with genes from other *Gynochothodes* species available in the NCBI database. It was found that the genes of *G. nanlingensis* differ from the reference genome and mapped sequences, raising questions about the identification of this species. In addition, the divergence time tree was constructed using fossil data (Figure S2) [29]. The estimated divergence times show connections with the RSCU values presented in Figure 5. The RSCU values of GCC and TTC decrease in clades that diverged later. For example, most species from the tribe Rubieae, which diverged later than the genus *Leptodermis*, have lower RSCU values. In the case of GCT, GGT, and TAG codons, RSCU values increase in taxa that diverged later.

**Figure 6:**
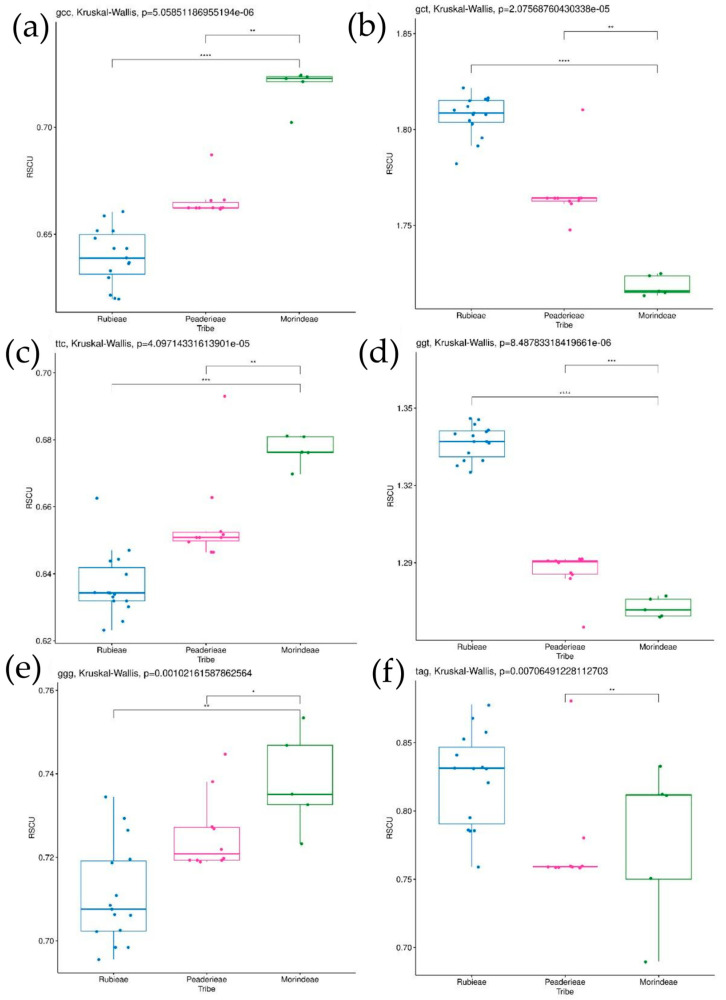
Boxplots show post-hoc Dunn test results for RSCUs of chosen codons between tribes Rubieae, Paederieae, and Morindeae. Stars indicate which groups differ significantly: one star—low differentiation, two stars—moderate differentiation, three stars—strong differentiation, and four stars—very strong differentiation (any values do not overlap between compared groups). Chosen codons are presented accordingly: (**a**) GCC, (**b**) GCT, (**c**) TTC, (**d**) GGT, (**e**) GGG, and (**f**) TAG.

The statistical analysis showed significant differences between Rubieae and Morindeae in the codons GCC, GCT, TTC, GGT, and GGG (Figure 6a–e, Table S15). None of these codons showed statistical differences between Rubieae and Paederieae, but all codons significantly differed between Paederieae and Morindeae. The TAG codon is very interesting because the RSCU value is very dynamic between genera, so a larger number of genera in Rubieae and Morindeae may influence the evolutionary trend shown by RSCU, making this trend random (Figure 6f).

**Figure 7:**
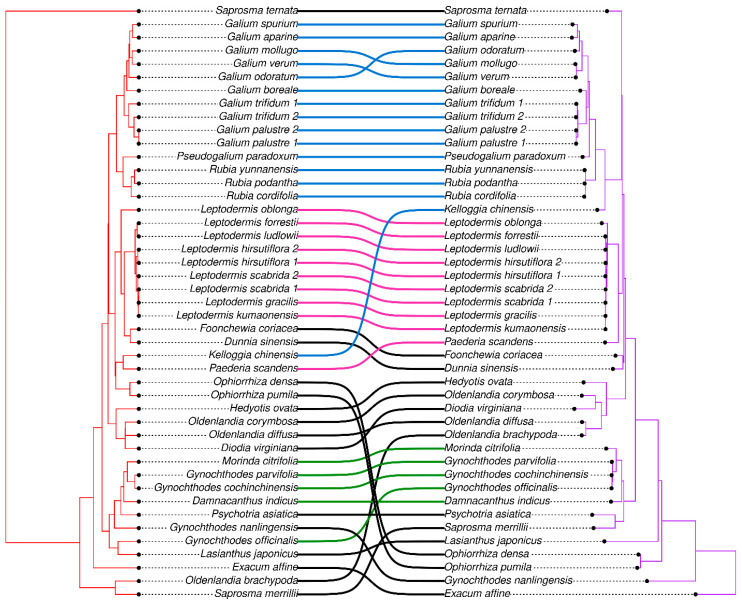
Comparison of tree based on RSCU (red tree) and a phylogenetic tree based on whole chloroplast genomes (purple tree). Lines between trees connect names of the same species to show the difference in the trees’ topology. Blue lines connect species from the tribe Rubieae, pink lines connect species from the tribe Paederieae, and green lines connect species from the tribe Morindeae. Black lines connect species that were not used in the RSCU comparison with phylogenetic relationships.

RSCU clustering is a method which allow scientists to visualise the Euclidean distance (calculated from RSCU values) between species (Figure 7). Usually, cluster trees based on RSCU do not reflect phylogenetic trees. However, in the case of the tribes Rubieae and Paederieae, the relationships between clusters and clades are similar, and they mostly differ between interspecies relationships. In the case of the tribe Rubieae, *Kelloggia chinensis* is the only species which left the Rubieae cluster. RSCU clustering showed a close relationship of this species with *Paederia scandens*, which did not group with the tribe Paederieae. The Morindeae group also differed, in the tree comparison, with one species—*Gynochthodes officinalis*—which grouped outside the Morindeae cluster.

## 4. Discussion

Except for a few exceptions, the gene content of the chloroplast genome is stable across *Galium*. All species have a quadripartite structure without clear differences between LSC, SSC, and IR. Early studies on the plastome of *Galium* sp. indicated that the *infA* gene likely has been transferred to the nuclear genome [38]. The absence of this gene was confirmed in the case of newly sequenced *G. trifidum* and *G. palustre* plastomes, but *G. verum* and *G. odoratum* plastomes contain this gene, as well as *Galium mollugo* (NC_036970). Pseudogenized forms of *infA* caused by frameshift have been found in partially sequenced *G. odoratum* and in *G. aparine* (NC_036969) [39]. The *infA* gene is present in the recently published chloroplast genome of *Galium spurium*, which additionally has four more transfer RNA genes than *G. trifidum* [40]. The number of protein-coding genes among species belonging to the Rubieae tribe varies from 79 (*Rubia yunnanensis*) to 87 (*K. chinensis*), and the number of tRNA genes from 30 (*R. yunnanensis* and *Galium* sp.) to 37 (*K. chinensis*) [41,42]. The GC content of the plastid genome seems to be stable in Rubioideae, ranging from 36.98% in *R. yunnanensis* to 37.1% in *K. chinensis*, showing no clear evolutionary pattern. Important data derived from the analysis of cpDNA are aggregations of changes between species of specific genera. The π diversity is one of the parameters used to measure such differences. Thirteen *Leptodermis* plastomes have shown nucleotide diversity hotspots in LSC: *trnS*—*trnG*, *rps2*—*rpoC2*; IR: *ycf2*—*ndhB*; and SSC: *ndhF*, *rpl32*—*ccsA*, *ccsA*—*ndhD*, *ndhA* [43]. The highest peak of the *Leptodermis* samples in our analysis was located very close to the *rps2*—*rpoC2* region, while between *trnS* and *trnG*, our plot turned out to be very low. Other hotspots similar to our results are close to *ycf1*—*ndhF*, *ndhF*, and *ccsA*. The peak in *rps2* is covered by a peak in *Galium* individuals, and the peak inside the two genes (*ycf1* and *ndhF*) is covered by both the *Galium* and *Rubia* genera. The highest points of nucleotide diversity in *Galium* coincide with the largest hotspots of SNPs and indels in *ycf1* and the *trnD*-GUC—*trnE*-UUC region. These regions are very distinct in comparison to *Rubia* and *Leptodermis*, but matching hotspots can be found in all the genera mentioned: the beginning of *rps16*, *trnT*-UGU—*trnL*-UAA, *ycf1*—*ndhF*, and *ccsA*. Regions that show high nucleotide diversity in other Rubiaceae genera are, e.g., in *Ophiorrhiza*, *petA*—*psbJ*, *trnH*-GUG—*psbA*, *trnS*-GCU—*trnR*-UCU, *psbM*—*trnD*-GUC, and *ndhC*—*trnM*-CAU, and one of these regions is also highly variable in the *Galium* genus (*trnD*-GUC) [44].

Phylogenetic trees based on complete chloroplast genomes usually show strong clade support in their bootstrap values [45]. The sequencing of complete plastid genomes is still a relatively laborious approach, and for this reason, plastome-based trees tend to have fewer species than those derived from single markers. Two trees (parsimony and Bayesian) based on three chloroplast regions: *rpoB*—*trnC*, *trnC*—*psbM*, and *trnL*—*trnF*—*ndhJ*, included most of the species used in our research (except *G. trifidum*), but their bootstrap values were low in the parsimony tree [45]. However, their Bayesian posterior probability (PP, >0.95) values resolved questionable relationships generated by the first tree. [45]. Both trees show similar relationships for *Galium* species used in our analysis (Figure 5). Other works confirmed the non-monophyly of the *Galium* genus, which is formed of clades that place *Asperula* species between *Galium* species [8,45,46]. The *Galium* relationships presented by our phylogram correlate with trees generated in other studies [8,45,46].

Except for single nuclear and chloroplast regions, and whole chloroplast genomes, the target enrichment method was used in the Rubiaceae family to analyse phylogenetic relationships [47]. The analysis of the Rubiaceae phylogeny using exonic regions obtained by target enrichment sequencing resulted in a well-resolved phylogenetic tree. This method also performed well in the Cinchonoideae and Ixoroideae subfamilies, confirming that target enrichment sequencing is a powerful tool in phylogenetic relationship analyses, especially when it is difficult to obtain good-quality molecular data from herbarium specimens [48].

Nuclear genes are often used for phylogenetic analysis separately or alongside chloroplast genes [49]. Nuclear genes can provide highly resolving phylogenetic trees. For instance, phylogenetic trees based on huge nuclear gene sets have shown 100% resolving force for Rubiaceae species [50]. Although the number of Rubiaceae species is much smaller than in our study, the phylogenetic relationships remain similar.

Other problems with the Rubiaceae family are the occurrence of non-monophyletic genera, e.g., *Saprosma ternatum* (*S. ternata*) appeared in the phylogenetic tree far away from *S. crassipes*, which was grouped with *Lithosanthes biflora* and *Lasianthus* species [51]. On the other hand, the genus *Gynochthodes* is usually monophyletic, which was not confirmed by our phylogenetic tree like in the case of the *Saprosma* genus [52,53]. Further analyses and the usage of new methods are needed to ensure that *Saprosma* and *Gynochthodes* are non-monophyletic. In the case of *Galium*, it is not possible to confirm that this genus is non-monophyletic with whole chloroplast genomes because plastomes of *Asperula* species are needed.

The relative synonymous codon usage (RSCU) in chloroplast genomes shows a preference for using specific codons that might be a result of natural selection or mutational pressure [54]. Evolution might affect chloroplast genomes by changing their structure, content, and creation of nucleotide differences. Most plastome codons end with A or T (we are using a thymine instead of uracil for a convenience because our analysis is based on DNA material), which might be an effect of many million years of development of chloroplast genomes [43,54,55,56,57,58]. However, the RSCU is sometimes very similar in the case of specific codons. Our results are consistent with a Theaceae species investigation, which describe a preference of AGA, GCT, and TTA codons (RSCU > 1.8) for arginine, alanine, and leucine, respectively [59]. Moreover, the use of a bigger data set in our study than the one used in the Theaceae species analysis allowed for the observation that RSCU might change during the evolution of plant groups. Although, the correlation of RSCU with phylogeny is not clear. The comparison of RSCU distribution and the CDS-based phylogenetic tree of *Prunus* species has shown clustering inconsistencies [60]. Our analysis also showed differences between RSCU clusters and phylogenetic clades, but in the case of the tribes Rubieae and Paederieae, the species composition of clusters differ only with one species: *K. chinensis* in the case of Rubieae and *P. scandens* in the case of Paederieae. Furthermore, these species appear to be very similar in terms of RSCU.

The organellar genomes of Rubiaceae species have never been used as superbarcodes, and scientists have mainly worked with nuclear DNA fragments such as internal transcribed spacers (ITS) and single genes or regions of plastome: *matK*, *trnH*—*psbA*, *rbcL*, *rps16*, *ndhF*, *petD*, and *trnT*—*trnF* [60,61,62,63]. Normally, superbarcodes should be diverse enough to distinguish interspecies differences, so the mitochondrial genome is very rarely used in plant species delimitation. However, the mitogenomes of *Calypogeia* species can be used as superbarcodes in super-mitobarcoding [64]. The highest condensations of diagnostic nucleotides were found within the most variable regions such as spacers: *nad2*—*rps12* and *nad3*—*nad7*, pseudogenes (*nad7*) or genes (e.g., *rpl2*). It is interesting that in other work, the plastid genome of *Calypogeia* species appeared to be less diverse than its mitogenome [13]. The largest aggregations of diagnostic nucleotides were found at window positions 30,000 and 55,000. In another work on liverwort species, terrestrial and water forms of *Apopellia endiviifolia* were compared in terms of their plastomes; their diagnostic nucleotides were much more numerous in this case than in the *Calypogeia* plastome, and the chloroplast genome was able to separate two different forms of *Apopellia* [65]. One of the most diagnostic regions in *Apopellia* was *ycf*1, which performs well as a DNA barcode in vascular plants [66]. However, our analysis shows that *ycf*1 is quite weak for species delimitation in all three genera (*Galium*, *Rubia*, *Leptodermis*), despite the high nucleotide diversity in this region. Most studies concerning *Galium* genus use other plastid regions that enable the separation of species in the phylogenetic tree, e.g., *atpB*—*rbcL*, *rpoB*—*trnC*, *trnC*—*psbM*, *trnL*—*trnF*—*ndhJ*, *rps16*, and *rpl32*—*trnL* [8,45,46]. Most of these regions performed well in our species delimitation, except *atpB*—*rbcL* and *rpoB*—*trnC*. Additionally, the best region we found was *trnE*-UUC—*psbD*, which is not mentioned in any phylogenetic studies of the *Galium* genus. Many scientists focus on mainstream genes in their studies, and they can omit regions that are crucial for a specific taxonomic group. For instance, the *trnL*—*trnF* region, the *petD* gene, and two nuclear markers were previously successfully used for species delimitation of the *Chiococceae* tribe (Cinchonoideae, Rubiaceae) [67]. The *trnL*-UAA—*trnF*-GAA region creates a clear peak of diagnostic nucleotides followed by high nucleotide diversity in *Galium*, and it might provide valuable information for molecular studies of this genus. The overwhelming number and diversity of organisms included in the Rubiaceae family forces botanists to find new molecular markers that allow for the successful identification of species. Comparisons of our results show that *Galium* and *Rubia* have similar regions that, together, effectively separate species. Research about the *Leptodermis* genus showed six variable regions that could be used as potential cpDNA markers: *trnS*—*trnG*, *rps2*—*rpoC2*, *ycf2*—*ndhB*, *ndhF*, *rpl32*—*ccsA*, *ccsA*—*ndhD*, *ndhA* [43]. The diagnostic nucleotides calculated by us show that a characteristic concentration is located only in the *trnS*-GCU—*trnG*-GCC region that can be used as a potential molecular marker. This region has previously been used to resolve phylogenetic relationships inside the Ixoroidae subfamily [49].

## 5. Conclusions

This research enabled us to obtain insights into four never-described chloroplast genomes of *G. trifidum*, *G. palustre*, *G. odoratum*, and *G. verum*. These genomes were compared with other available *Galium* plastomes, revealing regions with potential barcodes. Moreover, the relative synonymous codon usages of particular codons showed clear differences between three different tribes: Rubieae, Paederieae, and Morindeae. New chloroplast genomes might be useful in future phylogenetic studies, along with superbarcoding and molecular delimitation, because the region *trnE*-UUC—*psbD* appears to be a new potential genetic marker for the genus *Galium*.

## Data Availability

All newly generated chloroplast genomes are deposited in the GenBank database under the following accession numbers: *G. palustre* 1: NC_082336, *G. palustre* 2: OQ434191, *G. trifidum* 1: NC_082337, *G. trifidum* 2: OQ434193, *G. verum*: NC_082338, *G. odoratum*: NC_082335.

## Supplementary Materials

The following supporting information can be downloaded at: https://www.mdpi.com/article/10.3390/genes15050562/s1, Table S1: The studied plastomes, Table S2: The origin of the assembled genomes, Table S3: Plastome differences, Table S4: SNPs per 100 bp, Table S5: SNPs per region, Table S6: Indels per 100 bp, Table S7: Indels per region, Table S8: Nucleotide (π) diversity, Table S9: SNP % in coding regions, Table S10: Indel % in coding region, Table S11: SNP % in noncoding regions, Table S12: Indel % in noncoding regions, Table S13: Diagnostides per 100 bp, Table S14: RSCU, Table S15: post hoc table for selected species, Figure S1: The alignment of *ycf*1 pseudogene and *ycf*1 functional gene, Figure S2: The divergence time tree. File S1: The FASTA alignment.
