# Supplementary figures and images for: Decoding Evolution of Rubioideae: Plastomes Reveal Sweet Secrets of Codon Usage, Diagnostides, and Superbarcoding
Source: Genes (Basel). 2024 Apr 27;15(5):562. doi: 10.3390/genes15050562 (PMC11121115; doi:10.3390/genes15050562)

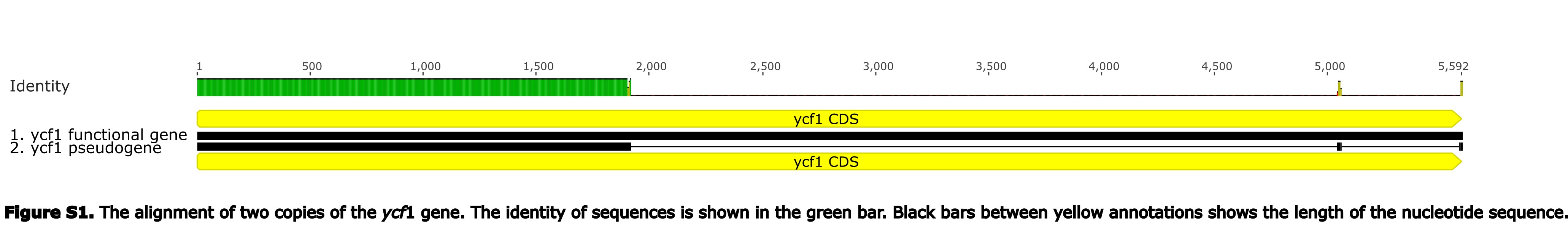

Supplement: Supplementary file 1 [file genes-15-00562-s001.zip › supplementary/Figure S1.jpg]

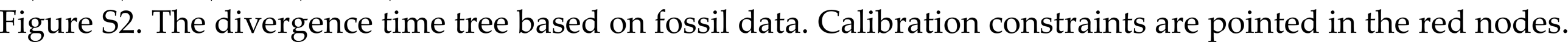

Supplement: Supplementary file 1 [file genes-15-00562-s001.zip › supplementary/Figure S2.pdf]
